# Music Therapy in the Psychosocial Treatment of Adult Cancer Patients: A Systematic Review and Meta-Analysis

**DOI:** 10.3389/fpsyg.2020.00651

**Published:** 2020-04-16

**Authors:** Friederike Köhler, Zoe-Sofia Martin, Ruth-Susanne Hertrampf, Christine Gäbel, Jens Kessler, Beate Ditzen, Marco Warth

**Affiliations:** ^1^Institute of Medical Psychology, Center for Psychosocial Medicine, University Hospital Heidelberg, Heidelberg, Germany; ^2^Ruprecht-Karls University Heidelberg, Heidelberg, Germany; ^3^Department of Communication and Psychology, Aalborg University, Aalborg, Denmark; ^4^Center of Pain Therapy and Palliative Care Medicine, Department of Anesthesiology, University Hospital Heidelberg, Heidelberg, Germany

**Keywords:** music therapy, oncology, cancer, effectiveness, randomized controlled trials, quality of life, supportive care, complementary therapies

## Abstract

**Introduction:** Music therapy is used as an adjunct oncological treatment aiming at the improvement of psychological and physical well-being through music. A growing body of randomized and non-randomized controlled trials has been published and reviewed recently. However, a global, quantitative assessment of the effectiveness of music therapy in adult cancer care is missing. The present study thus aims to synthesize the evidence of music therapy in different oncological treatment phases.

**Methods:** We conducted a pre-registered systematic review and meta-analysis (PROSPERO-ID: CRD42019133084) following standard guidelines. We searched electronic databases for studies on music therapy performed by a therapist with adult cancer patients.

**Results:** The narrative synthesis included thirty studies showing that music therapy overall had positive effects on a broad range of outcomes, with techniques and effects varying in different phases. During curative treatment, results were most promising with regard to anxiety, depression, and pain medication intake, while in palliative settings, improvements with regard to quality of life, spiritual well-being, pain, and stress were reported. Twenty-one studies were included in the meta-analysis which showed small but significant effects of music therapy on psychological well-being (*d* = 0.35, *p* < 0.001), physical symptom distress (*d* = −0.26, *p* = 0.017), and quality of life (*d* = 0.36, *p* = 0.023). Heterogeneity between effect sizes was small to medium. Moderator analyses identified studies with a single session of music therapy and the use of receptive techniques to produce larger effects regarding psychological well-being.

**Conclusion:** Music therapy can improve relevant health-outcomes in cancer patients and should therefore be offered in various treatment phases. Future research should include potential moderators such as individual information about patients to find out who benefits most from different kinds of music therapy.

## Introduction

Music therapy is a frequently used complementary and creative arts treatment in psychosocial cancer care (Bro et al., 2018). The general goal is to relieve symptom distress and to improve quality of life of patients in various stages of an oncological disease. Particularly in advanced cancer populations and palliative care, music therapy has recently received high attention in both research and clinical care (Stützlinger et al., 2018; Warth et al., 2019a). The use of music and sounds to affect human spirit and to heal dates back to ancient times. However, the modern understanding of clinical music therapy as a psychotherapeutic health-care discipline has developed after World War II, when academic courses and trainings as well as national associations were established around the world (Edwards, 2008). Today, music therapy is defined as “the systematic use of music within a therapeutic relationship which aims at restoring, maintaining and furthering emotional, physical and mental health” (Deutsche Musiktherapeutische Gesellschaft, 2019). Highlighting the importance of the therapeutic relationship, this definition clearly distinguishes music therapy from music medicine or mere listening interventions, such as listening to prerecorded music during surgery (Bradt et al., 2016). Nowadays, various health-care settings, including psychiatry, geriatrics, palliative care and oncology, offer music therapy to promote psychological, physical, or spiritual well-being (Deutsche Musiktherapeutische Gesellschaft, 2019).

In psycho-oncological care, music therapy is recommended by national guidelines in Germany as a treatment option to alleviate anxiety or existential fears (Leitlinienprogramm Onkologie., 2014) In the course of a life-threatening illness and its treatment, patients and relatives can experience a multitude of physical, psychological, social and spiritual distress strongly impairing their quality of life (Holland et al., 2010). Therefore, a multi-professional oncological treatment team is necessary to provide both medical and non-pharmacological interventions to address the patient's diverse needs. Psychosocial interventions, like psychotherapy or creative arts therapies, particularly aim at improving psychological well-being, building coping skills, and establishing social resources (Holland et al., 2010).

For this purpose, music therapy specifically can use music as a way of non-verbal expression and communication of cancer patients. Particularly, music therapists in oncology can offer multifaceted support in dealing with anxiety related to the disease or the medical procedures, in coping with stressful physical and emotional conditions, in stabilizing mood fluctuations, and in symptom management (e.g., pain, dyspnea, fatigue). Moreover, music therapy has been used to facilitate communication in patients and relatives as well as to address spiritual needs and existential fears (Bradt et al., 2016).

Moreover, music therapy was shown to promote relaxation and thus reduce stress, respiratory problems, and pain (Stützlinger et al., 2018). To achieve these goals, music therapists can offer a wide range of techniques to cancer patients and mostly tailor their therapeutic program to the individual's needs. Generally, active techniques can be distinguished from receptive techniques. In active music therapy, the patient participates in the production of music (e.g., by singing or playing an instrument), whereas receptive music therapy guides the patient in listening to live or recorded music (Stützlinger et al., 2018). In the treatment of cancer patients, techniques mainly encompass music-assisted relaxation and imagery, songs and improvisations (Warth et al., 2015b).

Previous reviews and meta-analyses of music interventions in oncology have shown positive effects on anxiety, depression, fatigue, and pain as well as physiological parameters (heart rate, respiration rate, and blood pressure) in cancer patients (Boyde et al., 2012; Zhang et al., 2012; Archie et al., 2013; Nightingale et al., 2013; Tsai et al., 2014; Bradt et al., 2016; Bro et al., 2018; Stützlinger et al., 2018; Gramaglia et al., 2019). However, the validity of these reviews was often restricted with regard to the inclusion or exclusion of certain patient populations, outcomes or interventions, which impeded a general conclusion about the quantitative effect of music therapy in adult cancer patients. For instance, one review was limited to evidence available in Chinese (Zhang et al., 2012), while other meta-analyses included studies with underage cancer patients (Boyde et al., 2012; Bradt et al., 2016), which from a clinical point of view requires a considerably distinct therapeutic approach. Another recent meta-analysis (Bro et al., 2018) included only cancer patients who underwent curative treatment while studies investigating patients in other treatment phases (e.g., during diagnoses, rehabilitation, or palliative care) were excluded. Similarly, some reviews focused solely on palliative care (Archie et al., 2013; Gramaglia et al., 2019) or included studies with music medicine interventions (Nightingale et al., 2013; Tsai et al., 2014).

A recent health technology assessment in Germany (Stützlinger et al., 2018) specifically considered music therapy interventions with more than one session attended by full age cancer patients in all treatment phases and concluded that music therapy has a positive short-term effect on psychological outcomes in cancer patients. No evidence was found for long-term or physiological effects. However, these results are based on a systematic review without meta-analytical support. Therefore, the aim of the present review and meta-analysis was to provide a narrative and quantitative synthesis of the effects of music therapy in adult cancer patients in all stages of the disease.

## Materials and Methods

The review was conducted in accordance with the Preferred Reporting Items for Systematic Review and Meta-Analyses (PRISMA) guidelines (Liberati et al., 2009). Additionally, we performed a meta-analysis with studies providing sufficient data. At the beginning of our research, a study protocol was published in the international prospective register of systematic reviews (PROSPERO-ID: CRD42019133084).

### Eligibility Criteria

Criteria for inclusion were pre-specified according to the PICOS framework (participants, interventions, comparisons, outcomes, study design) (Liberati et al., 2009) and can be seen in Table 1. Studies investigating adult patients with more than 80% of them having a primary cancer diagnosis of any type at any stage were included. Patients received either inpatient or outpatient oncological treatment. To meet the definition of music therapy, the interventions needed to be delivered by a trained therapist. Active as well as receptive interventions were included. Outcomes were not specified in the search syntax in order to get a complete list of variables measured in this context. Experimental groups could be compared to a waiting list group, a treatment as usual group or an active control group with a pretest-posttest-comparison. Randomized controlled trials as well as controlled clinical trials were accepted.

**Table 1:** Eligibility criteria.

**Domain**	**Inclusion criteria**
Patients	• Adult cancer patients at all stages • More than 80% with a primary cancer diagnosis
Interventions	• Music therapy provided by a trained therapist • Active and receptive interventions
Comparators	• Waiting list • Treatment as usual • Active control group
Outcomes	• Not specified
Study designs	• Randomized controlled trials • Controlled clinical trials

### Literature Search

Electronic sources for primary studies search were the databases PubMed, PsychInfo, and CINAHL. The syntax used for literature search in PsychInfo was:

(AB oncol^*^ OR AB cancer OR AB carcinomatosis OR AB cancer patients OR AB carcinosis OR AB palliative care OR AB leukemia OR AB carcino^*^ OR AB neoplasm OR AB chemotherapy OR AB end of life OR AB hospice OR AB tumor OR AB malign^*^) AND (AB drumming ORAB choir ORAB melody OR AB music OR AB singing) AND (AB random^*^ OR AB rct OR AB controlled trial OR AB cct OR AB clinical trial).

Additionally, relevant reviews concerning this topic and references of primary studies were hand-searched. Only studies published in English or German were included. The literature search was conducted in February 2019.

### Study Selection, Data Extraction, and Risk of Bias Assessment

Detected studies were imported into Rayyan (Ouzzani et al., 2016). Two researchers independently screened abstracts and rated them according to the eligibility criteria. In case of exclusion, the most prominent reason was given. Discrepancies were resolved by discussion. Afterwards, full texts were read and if in line with eligibility criteria, data was extracted from the selected studies and entered into a coding sheet containing the PICOS categories and the Cochrane Risk of Bias Assessment Tool (Table 2) which helps to assess methodological study quality and was adopted for psychotherapy research (Munder and Barth, 2017). Again, two researchers rated independently and discussed discrepancies.

**Table 2:** Study characteristics.

**Study**	**Setting and patients**	**Intervention**	**Control group**	**Study design**	**Main findings**	**Outcomes included in meta-analysis**
Alcântara-Silva et al. (2018)	Outpatient cancer treatment hospital, patients with gynecological cancer, *N* = 164	Music listening; 10 sessions, 35 min	TAU	RCT	Stronger increase in quality of life, stronger decrease in fatigue and depression in EG	
Allen (2010)	After care, patients with breast cancer who were in remission, *N* = 11	Music imaging (group therapy); 10 sessions, 60 min	ACG: cognitive behavioral therapy	RCT	Stronger increase in feelings of identity, family role relationship, self-esteem and body image in EG	
Bates et al. (2017)*	Inpatient blood and marrow transplantation unit, patients with lymphoma or myeloma, *N* = 82	Music listening and creating; 2 sessions, 30 min.	TAU	RCT	Less pain medication in EG, no significant difference in subjective pain perception	PSYCH: POMS
Bieligmeyer et al. (2018)*	Inpatient oncology department, patients with breast or colorectal cancer, *N* = 44	Sound bed; 1 session, 15 min	TAU	RCT	Stronger increase in well-being, quality of life and physiological symptoms in EG, no significant differences in pain and social extraversion	PSYCH: BMQ, PHYSIC: VAS
Bradt et al. (2015)*	In and outpatients from a hospital, patients with cancer (various types), *N* = 31	Music therapy (multiple techniques); 2 sessions, 20–45 min	ACG: listening to music	RCT	No significant group differences in mood, anxiety, relaxation and pain	PSYCH: VAS, PHYSIC: 11-point numeric rating scale
Burns (2001)*	Outpatient oncology offices, patients with breast or ovarian cancer, *N* = 8	Bonny method of guided imagery and music; 10 sessions, 60–90 min	Waitlist	RCT	Stronger increase in mood and quality of life in EG	PSYCH: POMS, QOL: QOL-CA
Burns et al. (2007)	Inpatient hematology oncology unit, patients with leukemia	Music imagery; 8 sessions, 45 min	TAU	RCT	No significant group difference in positive and negative effect, anxiety and quality of life	
Burns et al. (2018)	Outpatients from cancer centers, patients with various types of cancer, *N* = 86	Guided visualization with music; 1 session, 45–50 min	ACG: listening to music	RCT	Stronger increase in responsiveness and benefit finding in EG, stronger decrease in distress in CG	
Cassileth et al. (2003)*	Inpatient cancer centers, patients with lymphoma or myeloma, *N* = 69	Music listening and creating; 3–7 sessions, 20–30 min.	TAU	RCT	Stronger decrease in depression, anxiety and mood in EG	PSYCH: POMS
Chen et al. (2018)	Outpatient medical center, patients with breast cancer, *N* = 52	Music imagery; 8 sessions, 60 min	TAU	RCT	Stronger decrease of depression, helplessness, hopelessness and cognitive avoidance in EG	
Cook and Silverman (2013)	Inpatient oncology-hematology unit, patients with leukemia and other cancers, *N* = 34	Music listening and conversations; 3 sessions, 15–30 min	Waitlist	RCT	Stronger increase in spiritual well-being in EG	
Domingo et al. (2015)*	Inpatient palliative care unit, patients with advanced cancer, *N* = 68	Music therapy (multiple techniques); 4 sessions, 30*40 min	TAU	CCT	Stronger increase in well-being in EG	PSYCH: HADS, QOL: well-being single item
Dóro et al. (2017)*	Inpatient allogeneic hematopoietic stem cell transplantation unit, patients with neoplastic hematologic disorders, *N* = 100	Music singing and improvisation; 8 session, 30 min	TAU	RCT	Stronger increase in mood in EG, no significant difference in anxiety and pain	PSYCH: VAS; PHYSIC: VAS
Fredenburg and Silverman (2014)*	Inpatient adult blood and marrow transplantation unit, patients with leukemia and lymphoma, *N* = 11	Music therapy (multiple techniques); 30–45 min	Waitlist	RCT	No significant group difference	PHYSIC: MFI
Fredenburg and Silverman (2014)	Inpatient adult blood and marrow transplantation unit, patients with leukemia, lymphoma and myeloma, *N* = 32	Music therapy (multiple techniques); 1 sessions, 30 min	Waitlist	RCT	Stronger decrease of pain in EG, stronger increase in negative and positive effect in EG	
Gutgsell et al. (2013)*	Inpatient medical center, patients with advanced cancer (26 non-cancer patients), *N* = 200	Music relaxation; 1 session, 20 min	Waitlist	RCT	Stronger decrease in pain in EG	PHYSIC: 11-point numeric rating scale
Hanser et al. (2006)*	In and outpatients from breast oncology clinic, patients with breast cancer, *N* = 42	Music therapy (multiple techniques); 3 session, 45 min	TAU	RCT	Increase in relaxation, comfort and happiness in EG	QOL: FACT-G, PHYSIC: FACT-G (subscale), PSYCH: HADS
Hilliard (2003)*	Outpatient hospice, patients with advanced cancer, *N* = 80	Music therapy (multiple techniques); 2–13 sessions	TAU	RCT	Stronger increase in quality of life in EG, no significant difference in length of life or physical functioning	PSYCH: HQLI-R, QOL: HQLI-R
Horne-Thompson and Grocke (2008)*	Inpatient palliative care unit, patients with advanced cancer (1 non-cancer patient), *N* = 25	Music therapy (multiple techniques); 1 session, 20–40 min	ACG: volunteer visit	RCT	Stronger decrease in anxiety	PSYCH: ESAS, PHYSIC: ESAS
Letwin and Silverman (2017)*	Inpatient medical oncology/hematology unit, patients with various types of cancer, *N* = 15	Music listening; 2 sessions, 30–45 min	Waitlist	RCT	No significant group difference in resilience and pain	PSYCH: RSES, PHYSIC: 11-point numeric rating scale
Lin et al. (2011)*	Inpatient medical center/hospital, patients with various types of cancer, *N* = 98	Music imagery; 1 session, 60 min	TAU	RCT	Stronger decrease in anxiety in EG, stronger increase in skin temperature in EG	PSYCH: STAI
Palmer et al. (2015)*	Inpatient university hospital, patients with (potential) breast cancer, *N* = 201	Music listening before and during surgery; 1 session	TAU	RCT	Stronger decrease in anxiety and faster recovery after surgery in EG	PSYCH: VAS
Porter et al. (2018)*	Inpatient palliative care unit, patients with advanced cancer (4 non-cancer patients), *N* = 42	Music therapy (multiple sessions); 2–6 sessions, 45 min	TAU	RCT	Stronger increase in well-being	QOL: MQoL, PSYCH: MQoL, PHYSIC: MQoL
Ramirez et al. (2018)	Inpatient palliative care, patients with advanced cancer, *N* = 40	Music relaxation, active and receptive songs; 1 session, 30 min	ACG: Conversation about music	RCT	Stronger increase in valence and arousal and well-being in EG	
Rossetti et al. (2017)*	Outpatient medical center, patients with breast or head and neck cancer, *N* = 78	Music therapy (multiple techniques); 1 session, 60 min	TAU	RCT	Stronger decrease in anxiety and distress in EG	PSYCH: STAI
Tuinmann et al. (2017)*	Inpatient medical center, patients with lymphoma, *N* = 66	Music playing, singing and listening; 8 session, 20 min.	TAU	RCT	Stronger decrease in need of analgesics and subjective pain perception in EG	QOL: EORTC QLQ-C30, PAIN: EORTC QLQ-C30
Verstegen (2016)*	Inpatient blood and marrow transplantation unit, patients with cancer, *N* = 10	Music listening and therapeutic dialogue; 2 session, 30–60 min	TAU	RCT	Stronger increase in hope in EG, no significant difference in pain	PSYCH: HHI, PHYSIC: 11-point numeric rating scale
Wang et al. (2015)	Inpatient cancer hospital, patients with lung cancer, *N* = 60	Music relaxation during surgery, music listening afterwards; 5 sessions, 60 min	TAU	RCT	Stronger decrease in anxiety, lower blood pressure and heart rate, less need for analgesics in EG	
Warth et al. (2015b)*	Inpatient palliative care, patients with cancer (2 non-cancer patients), *N* = 84	Music relaxation; 2 sessions, 30 min	ACG: pre-recorded mindfulness exercise	RCT	Stronger increase in relaxation and well-being and in high-frequency oscillation of the heart rate and stronger decrease in fatigue in EG, no significant difference in pain	QOL: VAS, PSYCH: EORTC QLQ C15-PAL, PHYSIC: VAS
Yates and Silverman (2015)*	Inpatient surgical oncology unit, patients with colon/rectal or uterine cancer, *N* = 26	Music listening; 1 session, 20–30 min	Waitlist	RCT	Stronger decrease in anxiety and increase in relaxation in EG	PSYCH: QMS

Studies marked with * were included in meta-analyses; TAU, treatment as usual; ACG, active control group; RCT, randomized controlled trial; CCT, controlled clinical trial; EG, experimental group; CG, Control group; PSYCH, psychological well-being; QOL, quality of life; PHYSIC, physical symptom distress; Poms, Profile of mood states; BMQ, Basler Mood Questionnaire; VAS, Visual Analogue Scale; QOL-CA, Quality of life – Cancer scale; HADS, Hospital Anxiety and Depression Scale; MFI, Multidimensional Fatigue Inventory; FACT-G, Functional Assessment of Cancer Therapy—General; HQLI-R, Hospice Quality of Life Index—Revised; ESAS, Edmonton Symptom Assessment Scale; RSES, Response to Stressful Events Scale; STAI, Stait Trait Anxiety Inventory; MQoL, McGill Quality of Life Questionnaire; EORTC QLQ-C30, Quality of Life Questionnaire; HHI, Herth Hope Index; EORTC QLQ-C15-Pal, Quality of Life Questionnaire for Palliative Care; QMS, 12-item quick mood scale.

Regarding the systematic review, all studies matching eligibility criteria were considered. Only studies providing sufficient data were then included in the meta-analysis. In case of missing information in the papers, authors were contacted by email. If they did not answer or could not provide the missing data, the study was excluded of the meta-analysis.

### Statistical Analysis

The list of outcome variables was grouped into categories. Three of them (psychological well-being, quality of life, and physical symptom distress) were applicable for meta-analysis (i.e., more than five effect sizes per category; pre- and post-means, sample sizes per study group, and standard deviations reported). Psychological well-being encompasses outcomes such as mood, anxiety, and depression, whereas physical symptom distress includes pain, generic physical symptom scales, and fatigue. Data of other categories (spiritual and social well-being as well as biomarkers) were only considered in the narrative synthesis. If a study reported multiple outcomes of one category, the measurement or kind of outcome that was used more often in other studies was included to avoid dependencies within categories.

Effect sizes were estimated based on the difference of mean pretest-posttest change in the treatment and control group divided by the pooled standard deviation before intervention. This calculation takes most advantage of the information given in the studies as it considers baseline differences between groups (Morris, 2008) and is recommended in a recent methodological review of meta-analyses in clinical psychology (Rubio-Aparicio et al., 2017). This effect size is a variation of Cohen's *d* and can be interpreted correspondingly (small: *d* = 0.2–0.5, medium: *d* = 0.5–0.8, large: *d* = 0.8) (Cohen, 1992). To calculate sampling variance of standardized mean change differences, the pretest-posttest correlation for quality of life, psychological well-being, and physical symptom distress was estimated based on previous research (Warth et al., 2015a).

Meta-analysis was performed using the “metafor” package in R (Viechtbauer, 2010). As it is implausible to assume that true effect sizes in all studies are the same due to variance e.g., in participants, interventions or study designs, a random effects model was computed. Heterogeneity was analyzed with τ, *Q*-test, and *I*^2^. τ describes the standard deviation of the true effect size, *Q* allows a significance test and *I*^2^ is the relation of true to observed heterogeneity (Borenstein et al., 2011). Plausible sources of heterogeneity were setting of cancer treatment (inpatient vs. outpatient/mixed), treatment phase (palliative vs. curative), type of music therapy (active/mixed vs. receptive), duration of music therapy session (short ≤ 30 min vs. long ≥ 30 min), frequency of music therapy sessions (one vs. more) and type of control group (treatment as usual/waiting list vs. active control). To find out if these possible moderators explained variance between effects, their values were dichotomized due to the small amount of studies in each category, and a moderator analysis was conducted.

To test presence of publication bias, funnel plots were inspected, and Egger's regression test for asymmetry was conducted (Egger et al., 1997). In case of asymmetry, the trim-and-fill method was applied to correct asymmetry in the funnel plot (Duval and Tweedie, 2000). In order to identify outliers and influential studies, Baujat plot was inspected and a set of diagnostics (e.g., standardized residuals, Cook's distance) was conducted. If an effect showed a pattern of deviation in graphical outputs of these statistics, it was excluded and sensitivity analysis was performed. Type I error probability was set at α = 0.05.

## Results

After the description of the study selection process, we provide a narrative synthesis of information from primary studies, categorized into different oncological treatment phases, and then proceed to the results of the meta-analysis. Table 2 presents an overview of the study characteristics. Of note, nine studies described in the narrative synthesis were excluded from statistical meta-analysis due to insufficient data reporting.

### Study Selection

**Figure 1:**
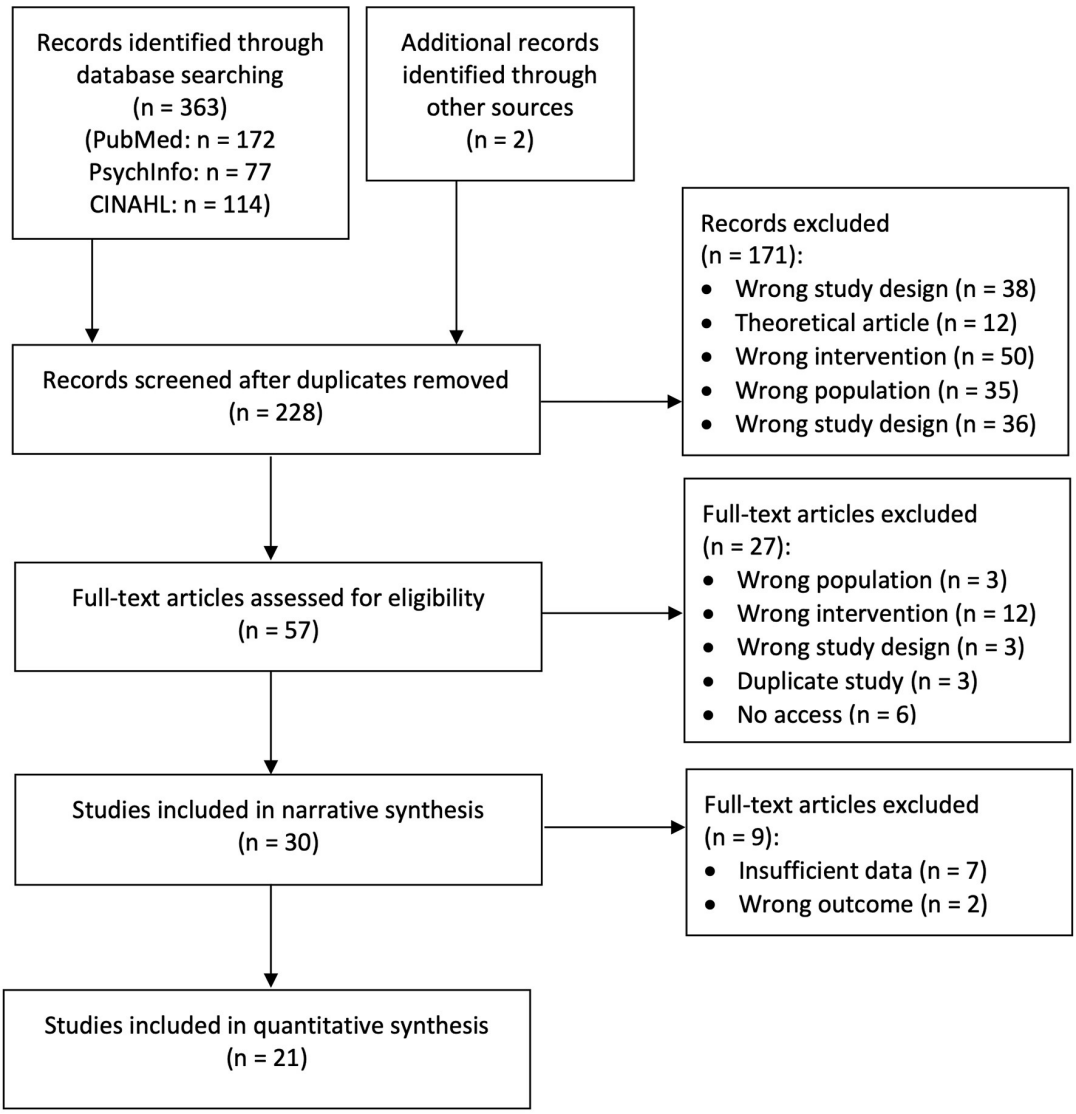
Flow Diagram of study selection. Studies with insufficient data and outcomes which did not fit into meta-analyses categories were not included in quantitative syntheses of results.

Data base search according to our syntax resulted in *n* = 363 studies. Two additional studies were identified through other sources. After removal of duplicates *n* = 228 studies remained. Their abstracts were screened and *n* = 171 studies were removed due to reasons listed in in Figure 1. Full texts of the remaining *n* = 57 studies were assessed for eligibility. After that *n* = 30 studies remained for the narrative synthesis. For quantitative analysis, studies with insufficient data (*n* = 7) as well as studies with measures that did not fit into one of the outcome categories (*n* = 2) (Cook and Silverman, 2013; Ramirez et al., 2018) were excluded and *n* = 21 studies remained. The full study selection process is shown in Figure 1.

### Risk of Bias Assessment

Results of risk of bias assessment can be seen in Table 3. Although ratings overall show high bias in all studies, the extent of different biases varies among studies. Only four studies out of 30 had low risk of bias in four categories (Palmer et al., 2015; Warth et al., 2015a; Letwin and Silverman, 2017; Bieligmeyer et al., 2018) and five in three categories (Cassileth et al., 2003; Hanser et al., 2006; Horne-Thompson and Grocke, 2008; Bradt et al., 2015; Dóro et al., 2017).

**Table 3:** Risk of bias assessment.

**References**	**RAND**	**ALLO**	**BLPP**	**BLOA**	**INCDAT**	**SELREP**	**TREAT**
Alcântara-Silva et al. (2018)	Low	Unclear	High	Unclear	Unclear	Unclear	Unclear
Allen (2010)	Low	Unclear	Unclear	Unclear	Unclear	Unclear	Low
Bates et al. (2017)	Low	Low	High	Unclear	Unclear	Unclear	Unclear
Bieligmeyer et al. (2018)	Low	Low	High	Unclear	Low	Low	Unclear
Bradt et al. (2015)	Low	Low	Unclear	Low	Unclear	Unclear	Unclear
Burns (2001)	Unclear	Unclear	High	Unclear	Unclear	Unclear	Unclear
Burns et al. (2007)	Unclear	Unclear	High	Unclear	Unclear	Unclear	Unclear
Burns et al. (2018)	Low	Unclear	Unclear	Low	Unclear	Unclear	Unclear
Cassileth et al. (2003)	Low	Low	High	Unclear	Low	Unclear	Unclear
Chen et al. (2018)	Unclear	Unclear	High	High	Low	Low	Unclear
Cook and Silverman (2013)	Low	Unclear	High	High	High	Unclear	Unclear
Domingo et al. (2015)	High	High	High	Unclear	Unclear	Unclear	Unclear
Dóro et al. (2017)	Low	Low	High	Low	Unclear	Unclear	Unclear
Fredenburg and Silverman (2014)	Low	Unclear	High	High	Unclear	Unclear	Unclear
Fredenburg and Silverman (2014)	Low	Unclear	High	High	High	Unclear	Unclear
Gutgsell et al. (2013)	Low	Low	Unclear	High	Unclear	Unclear	Unclear
Hanser et al. (2006)	Low	Low	High	Low	Unclear	Unclear	Unclear
Hilliard (2003)	Low	Unclear	High	High	Unclear	Unclear	Unclear
Horne-Thompson and Grocke (2008)	Low	Low	Unclear	Low	Unclear	Unclear	Unclear
Letwin and Silverman (2017)	Low	Low	High	High	Low	Low	Unclear
Lin et al. (2011)	Low	Unclear	High	High	Low	Unclear	Unclear
Palmer et al. (2015)	Low	Low	High	Unclear	Low	Low	Unclear
Porter et al. (2018)	Low	Low	High	High	High	Unclear	Unclear
Ramirez et al. (2018)	Low	Unclear	Unclear	Unclear	Unclear	Unclear	Unclear
Rossetti et al. (2017)	Low	Unclear	High	Unclear	Unclear	Unclear	Unclear
Tuinmann et al. (2017)	Unclear	Unclear	High	Unclear	Low	Low	Unclear
Verstegen (2016)	Unclear	Unclear	High	High	Unclear	Unclear	Unclear
Wang et al. (2015)	Low	Low	High	Unclear	Unclear	Unclear	Unclear
Warth et al. (2015b)	Low	Low	Unclear	High	Low	Low	Unclear
Yates and Silverman (2015)	Unclear	Unclear	High	High	High	Unclear	Unclear

*RAND, random sequence generation; ALLO, allocation concealment; BLPP, blinding of participants and personnel; BLOA, blinding of outcome assessors; INCDAT, incomplete outcome data; SELREP, selective outcome reporting; TREAT, treatment implementation*.

With regard to the specific categories, the risk in randomization and allocation sequence was low or unclear in all studies, except one that was a controlled clinical trial (Domingo et al., 2015). Risk of bias in blinding of participants and personnel was high or unclear because no study reported assessment of patients' expectancies. Five studies reported blinding of outcome assessors, the others did not provide sufficient data on outcome assessment or lacked blinding of outcome assessors. Four studies showed a high risk of inappropriate handling of missing data. No study was rated high on selective outcome reporting and four studies provided a study protocol (Palmer et al., 2015; Warth et al., 2015a; Tuinmann et al., 2017; Bieligmeyer et al., 2018). Only one study provided information on treatment implementation (Allen, 2010).

### Study Description and Narrative Synthesis

#### Music Therapy During Chemotherapy and Radiation

Ten studies examined the effects of music therapy in the course of chemotherapy or radiation treatment on diverse outcomes. Three focused specifically on music therapy as an adjunct treatment during chemotherapy. A study by Lin and colleagues investigated the effectiveness of three different study conditions in patients undergoing chemotherapy: A 1 h single music therapy session, a 30 min verbal guided relaxation, and standard care (Lin et al., 2011). Both music therapy and verbal relaxation showed to be effective in reducing chemotherapy-related anxiety compared to standard care alone. Besides, the music therapy group achieved a greater increase in skin temperature compared to the other groups. Hanser et al. (2006) compared three sessions of music therapy targeted at stress coping with standard care in breast cancer patients receiving chemotherapy. They detected positive effects on relaxation, comfort, and happiness, as well as on stress biomarkers (resting heart rate and blood pressure). A quasi-experimental trial studying the same population and cancer treatment found a reduction in depressive symptoms, helplessness, hopelessness, and cognitive avoidance after participation in a group music therapy program (Chen et al., 2018).

Two studies assessed the effects of music therapy in the course of radiation therapy and found reductions in anxiety and distress (Rossetti et al., 2017) as well as improvements regarding quality of life, fatigue, and depression (Alcântara-Silva et al., 2018). Techniques used in these studies encompassed listening to prerecorded music, live music therapy, and conversations with a therapist.

Five more studies were identified that studied patient populations receiving some sort of combination of chemotherapy and radiation treatment. Three of these were RCTs by Burns and colleagues that compared music-based imagery with different control conditions. In the first study, the guided music intervention led to greater improvements in mood states and quality of life than the waitlist control group (Burns, 2001). In the second study, the authors found improvements in both study groups regarding positive and negative affect as well as fatigue and anxiety (Burns et al., 2007). In the third study by this group, single sessions of music-based imagery attained higher responsiveness to music therapy and benefit finding, whereas preferred music listening led to lower distress (Burns et al., 2018). Fredenburg and Silverman (2014) conducted two pilot RCTs with post-transplantation patients receiving chemotherapy and/or radiation. The first study with a very low sample size found no between-group difference in fatigue compared to a waitlist control group (Fredenburg and Silverman, 2014). In contrast, the second study showed a single session of receptive music therapy with patient-selected live music to be effective with regard to reductions in negative affect and pain (Verstegen, 2016).

Hence, findings for music therapy as an adjunct treatment during radiation and chemotherapy are mixed, and allocation of studies to a certain primary treatment phase was partially ambiguous. Techniques used by music therapists were heterogeneous including relaxation and imagery, listening to music with a therapist, improvisation, and songwriting. Effects were most promising with regard to the reduction of anxiety, distress, depression, and pain.

#### Music Therapy During Surgery and Transplantation

Eight studies were included that focused on the use of music therapy in the course of surgery and transplantations. In one of them, lung cancer patients listened to relaxing music played by a therapist before and after surgery (Wang et al., 2015) and showed less anxiety, lower blood pressure, and heart rate parameters as well as a lower need for analgesics. Patients in Palmer and colleagues' study (Palmer et al., 2015) listened to a song before surgery and verbally reflected on it. During the operation, they listened to music chosen by the therapist. As a result, patients reported less anxiety and faster recovery in comparison to the control group. Yates and Silverman (2015) focused on the effect of music therapy after surgery and found a positive effect of receptive music therapy in combination with therapeutic conversation on anxiety and relaxation.

Another RCT studying patients undergoing blood and bone marrow transplantation (Verstegen, 2016) found no significant difference in pain but patients participating in two sessions of hope-oriented music therapy reported higher levels of hope than those in the control condition. Moreover, four studies examined the adjunct use of music therapy in the course of stem cell transplantations. In one study with two song-based music therapy sessions per week, greater improvements in mood, but no differences in self-rated pain were observed in comparison with the control group (Dóro et al., 2017). In another trial using non-standardized interventions, patients receiving two music therapy sessions required less pain medication even though the subjective pain reduction was similar in music therapy and the control group (Bates et al., 2017). The reduction in analgesics intake was also found in a similar study, where stem cell transplantation patients additionally reported a short-term reduction of subjective pain perception (Tuinmann et al., 2017). Moreover, positive effects of music therapy on immunological parameters and frequency of toxic side effects were found (Tuinmann et al., 2017). Another study investigated music therapy with stem cell transplantation patients over a longer period of time and with varying session numbers (Cassileth et al., 2003). The music therapy group showed positive effects on depression, anxiety, and mood in comparison to the control group.

Taken together, evidence on the use of music therapy during surgery and transplantation is promising, particularly with regard to the supportive management of anxiety and pain.

#### Music Therapy in Aftercare

In the only available pilot study on music therapy in oncological aftercare, successfully treated breast cancer patients took part in a group music therapy program over 10 weeks (Allen, 2010). Sessions encompassed receptive techniques like relaxation and imagery followed by debriefing, while the control group received cognitive behavioral psychotherapy. The small sample of 11 patients showed that patients in music therapy groups reported improved feelings of identity, family role relationship, self-esteem, and body image compared to the control group.

#### Music Therapy in Palliative Care

Seven trials were identified that studied palliative patient populations. One study (Warth et al., 2015a) investigated the effects of two sessions of guided music relaxation on psychological and physiological outcomes. The experimental group showed improvements in relaxation and well-being, an increase in heart rate variability and a reduction in fatigue in comparison with the active control group. Only with regard to pain, there was no significant group difference. In contrast, another study (Gutgsell et al., 2013) found an improvement in pain after one session of music relaxation with live music in palliative care compared to standard treatment. The effect of music relaxation was also supported by a study with electroencephalogram (EEG) data analysis (Ramirez et al., 2018). Patients in the music therapy group showed an increase in valence and arousal in comparison to the active control group which had loose conversations about music with the same music therapists. Moreover, questionnaires also indicated an improvement of psychological well-being through music therapy (Ramirez et al., 2018).

Other studies used a variety of techniques individually tailored to the patient's needs. For instance, in one study, terminally ill patients received at least two home visits from a music therapist (Hilliard, 2003). These patients reported a higher increase in quality of life than those with routine hospice service only. Regarding length of life or physical functioning, no differences were found. In another study using multiple, non-standardized techniques, a single session of music therapy was superior to a volunteer visit in reducing self-reported anxiety (Horne-Thompson and Grocke, 2008). A non-randomized trial also employing various techniques found the music therapy intervention to be superior with regard to well-being, anxiety, depression, and overall symptom distress compared to standard care alone (Domingo et al., 2015). A recent pilot RCT reported high patient drop-out and found improvements for existential well-being, but not for other quality of life domains in response to music therapy (Porter et al., 2018).

Hence, evidence regarding patients with a terminal disease in palliative care settings was mixed. Standardized treatments mainly focused on relaxation techniques while the majority of studies offered tailored interventions. Effects were most promising with regard to quality of life, pain, and spiritual well-being.

#### Music Therapy in Non-specified Treatment Phases

Four other studies examined the effects of music therapy on different outcomes in cancer patients, but did not specify a particular phase of cancer treatment. One study investigated a 2-days resilience-focused music therapy intervention. The authors found no significant group differences between the intervention group and the waitlist control group regarding resilience and pain (Letwin and Silverman, 2017). Another study compared music therapy and music medicine in cancer patients. They found both interventions to be equally helpful and supportive with regard to mood, anxiety, relaxation, and pain (Bradt et al., 2015). A third study examined a 10 min vibro-acoustic music therapy intervention vs. resting time within a cross-over-design. They reported immediate improvements in well-being, quality of life, and physiological changes. However, no group differences were found in pain and social extraversion (Bieligmeyer et al., 2018). The fourth study found positive effects of music therapy on spiritual well-being in comparison to standard care (Cook and Silverman, 2013). In this study, the music therapist played live music accompanied on the guitar.

### Quantitative Analysis

#### Meta-Analysis on Psychological Well-Being

**Figure 2:**
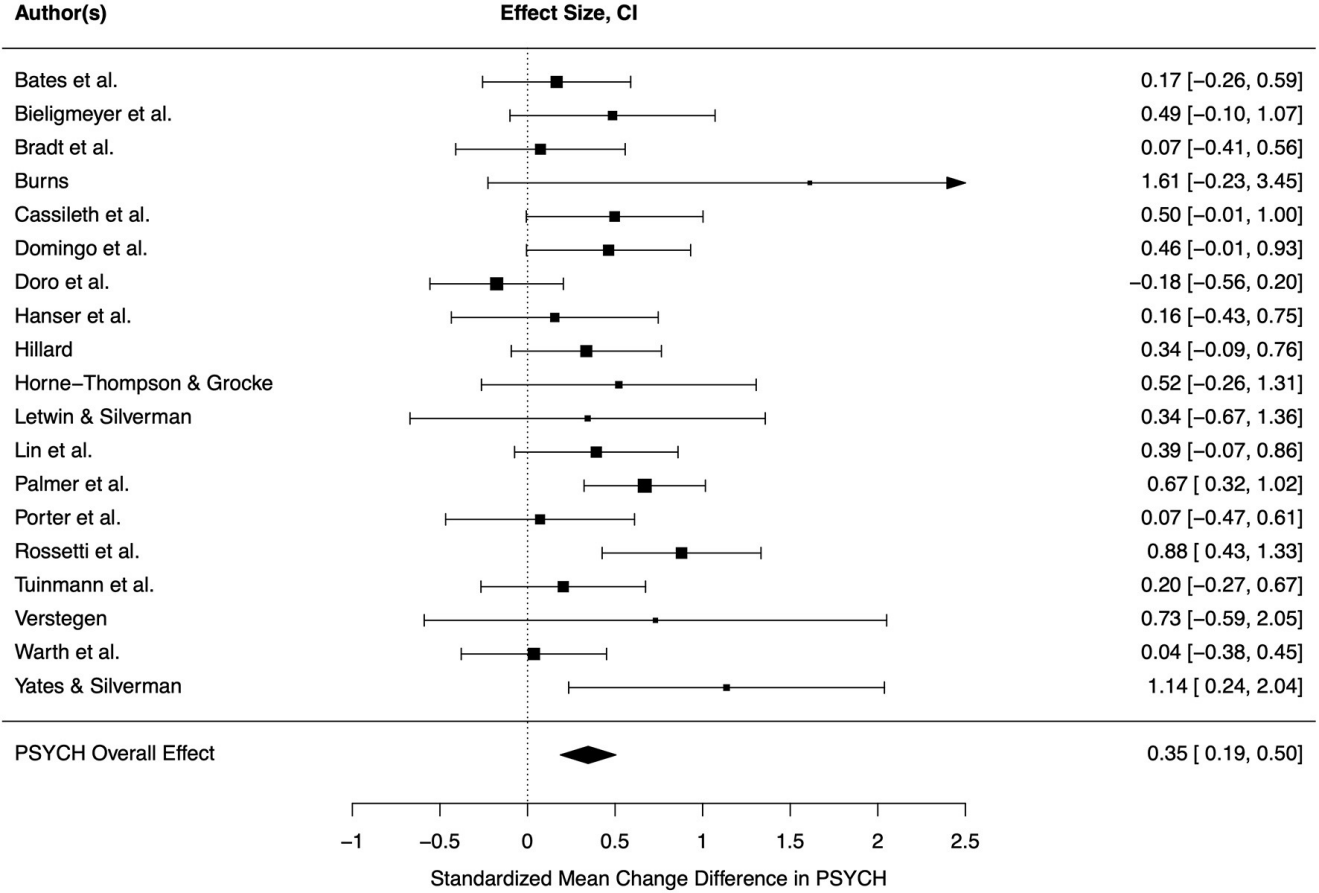
Forest plot for psychological well-being. CI, 95% confidence interval, PSYCH, psychological well-being.

Nineteen studies reported outcomes referring to psychological well-being. As mentioned above, one outcome per study was chosen for inclusion in our meta-analysis (*k* = 19). Included psychological outcomes encompassed anxiety, depression, psychological distress and well-being, mood, emotional functioning, relaxation, hope and resilience. Measurement instruments are shown in Table 3. Effect sizes of three primary studies indicated significantly better psychological well-being through music therapy in comparison to control group, other studies included zero in their confidence interval (Figure 2).

The overall effect calculated by the random-effects model was significant and small-sized (*d* = 0.35, CI = 0.19–0.50, *p* < 0.001), indicating that music therapy improves psychological well-being of oncological patients in comparison to control group treatment. Heterogeneity between studies was low and not significant (τ = 0.20, *Q* = 27.77, *p* = 0.066, *I*^2^ = 36.39%). Even though not necessarily indicated, moderator analysis was conducted, and frequency of music therapy sessions had a significant moderating effect (*F* = 15.79, *p* = 0.001). Thus, heterogeneity was lower and no longer close to significance (*Q* = 14.40, *p* = 0.639, *I*^2^ = 0.01%). Surprisingly, studies with a single session of music therapy showed greater improvements (*d* = 0.47) than therapy programs involving a higher number of sessions (*d* = 0.18). Moreover, the type of music therapy was a significant moderator (*F* = 6.20, *p* = 0.023) and contributed to the observed heterogeneity (*Q* = 20.06, *p* = 0.271, *I*^2^ = 22.59%). Receptive methods (*d* = 0.33) improved psychological well-being significantly better than active or mixed methods (*d* = 0.19). Other tested moderators did not explain variance across studies (*p* > 0.05). Egger's regression test was not significant (*p* > 0.05) indicating symmetry. However, visual analysis of funnel plot showed asymmetry. As a result of using trim-and-fill method, the effect size was smaller but still significant (*d* = 0.27, CI = 0.11–0.43, *p* = 0.001).

Inspection of Baujat plot and model diagnostics showed two highly influential studies (Dóro et al., 2017; Rossetti et al., 2017). One of them was the only negative effect size (Doro: *d* = −0.17, *n* = 100) and the other one had a large, positive effect size (Rossetti: *d* = 0.88, *n* = 78). Both analyzed a large sample size in comparison to the other studies. Exclusion of these studies did not lead to change in pooled effect size (*d* = 0.35, CI = 0.21–0.48, *p* < 0.001) but heterogeneity was lower (τ = 0.07, *Q* = 15.27, *p* = 0.505, *I*^2^ = 5.84%).

#### Meta-Analysis on Quality of Life

**Figure 3:**
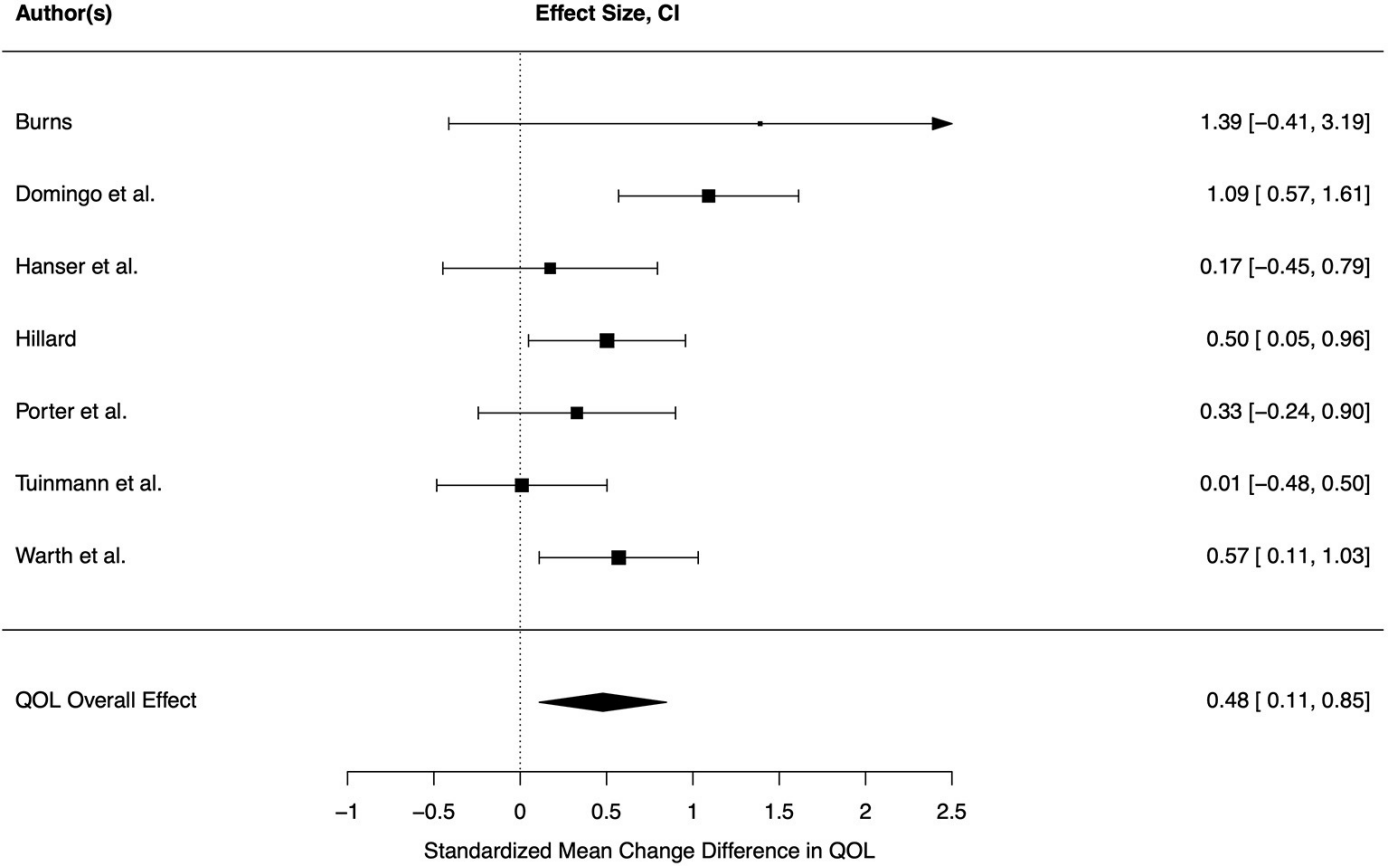
Forest plot for quality of life. CI, 95% confidence interval; QOL, quality of life.

Seven studies reported sufficient data on quality of life. Hence, *k* = 7 effect sizes were included in our analysis. Two measured general well-being, the other studies measured quality of live. Three single studies indicated significantly better quality of life in music therapy groups in comparison to control groups, the effect sizes of the other studies included zero in their confidence interval (Figure 3).

A small overall effect was statistically significant (*d* = 0.48, CI = 0.11–0.85, *p* = 0.019), indicating a positive effect of music therapy on quality of life in cancer patients in comparison to control conditions. Heterogeneity across effects was moderate but not significant (τ = 0.26, *Q* = 11.11, *p* = 0.085, *I*^2^ = 46.52%). The moderators were not able to explain variance across studies (all *p* > 0.05). Frequency of session was excluded as a moderator since all studies in this meta-analysis investigated more than one session. The funnel plot showed symmetry and Egger's regression test was not significant (*p* > 0.05), indicating absence of a publication bias. Based on Baujat plot and model diagnostics, one study with a large effect size (*d* = 1.09, *n* = 68) was identified as highly influential (Domingo et al., 2015). After exclusion of this study, the pooled effect size was lower, but still significant (*d* = 0.36, CI = 0.07–0.65, *p* = 0.023). Measurements of heterogeneity were all low after exclusion (τ = 0.00, *Q* = 4.73, *p* = 0.449, *I*^2^ = 0.00%) hinting at a large variance within studies due to small sample sizes. No moderator analysis was thus conducted. Funnel plot as well as Egger's regression test (*p* > 0.05) indicated no publication bias.

#### Meta-Analysis on Physical Symptom Distress

**Figure 4:**
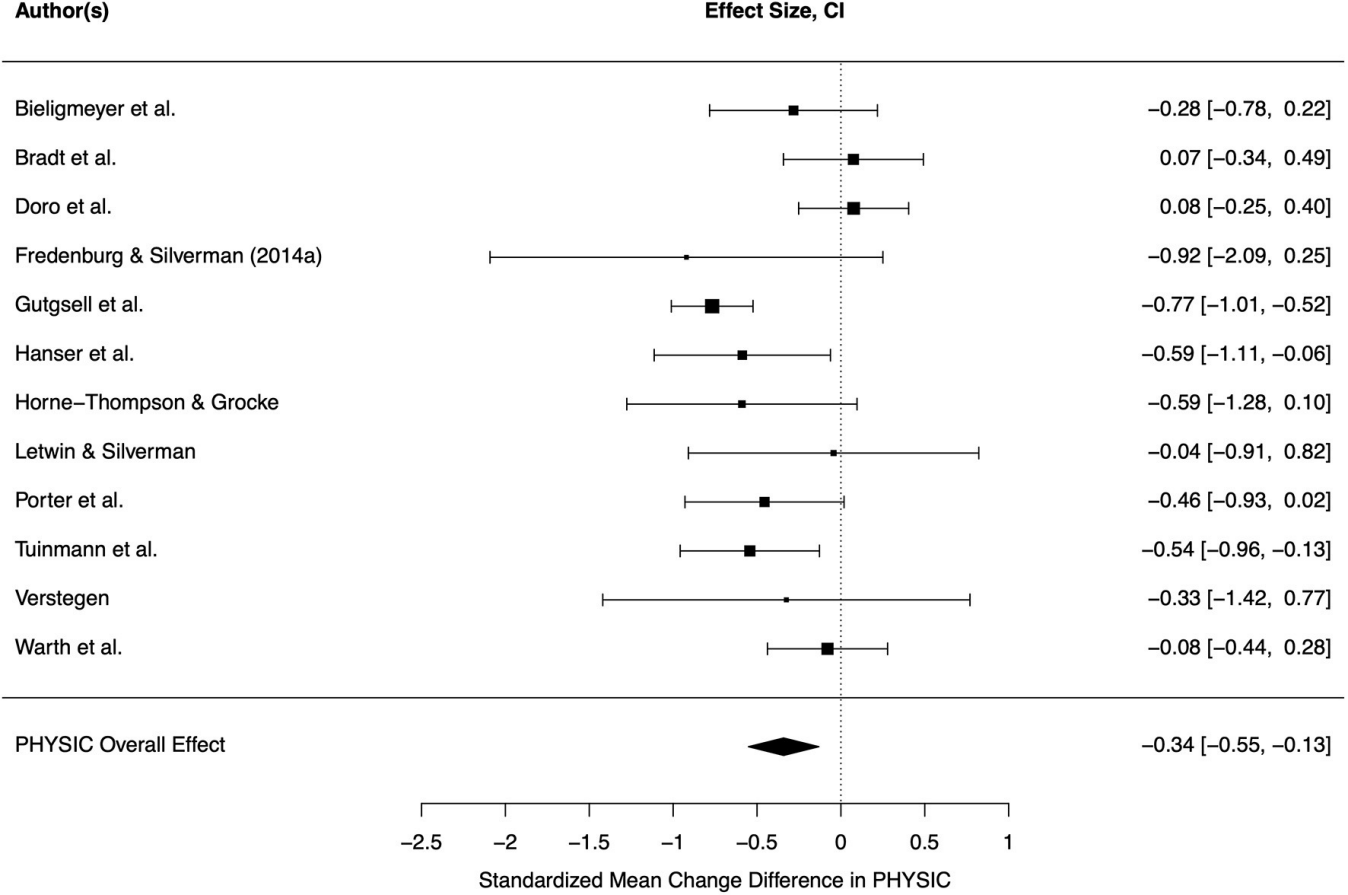
Forest plot for physical symptom distress. CI, 95% confidence interval; PHYSIC, physical symptom distress.

In the meta-analysis on physical symptom distress, *k* = 12 studies were included. Outcomes were pain (nine studies), physical well-being/symptoms (two studies) and fatigue. Measurement instruments are shown in Table 3. Three primary studies showed significant benefits of music therapy, while the other effect sizes did not differ significantly from zero (Figure 4).

The pooled effect was small-sized and significant (*d* = −0.34, CI = −0.55–0.13, *p* = 0.004). The included effects were heterogeneous (τ = 0.26, *Q* = 27.59, *p* = 0.004, *I*^2^ = 55.72%). Variance could not be explained by the tested moderators (all *p* > 0.05). Among these, frequency of sessions was closest to significance (*F* = 3.79, *p* = 0.080) and was able to contribute to overall heterogeneity (*Q* = 15.25, *p* = 0.123, *I*^2^ = 39.22%). Again, one session led to higher improvement of physical symptoms in comparison to control condition. Funnel plot showed symmetry of study distribution and Egger's regression test result supported this assumption (*p* > 0.05). Hence, there was no indication for a publication bias.

Inspection of Baujat plot and graphical outputs of model diagnostics pointed out one highly influential study (Gutgsell et al., 2013), which examined many participants (*n* = 200) in comparison to the other studies and found a medium-sized effect size (*d* = −0.77). Exclusion of this study led to a small decrease of effect size which was still significant (*d* = −0.26, CI = 0.06–0.46, *p* = 0.017). Heterogeneity among effects was no longer significant (τ = 0.18, *Q* = 13.54, *p* = 0.300, *I*^2^ = 33.09%). Hence, no further moderator analysis was conducted. Funnel plot as well as Egger's regression test (*p* > 0.05) indicated no publication bias.

## Discussion

In order to provide an overview of the impact of music therapy in adult cancer care, we conducted a systematic review and meta-analysis. We included only studies investigating music therapy performed by a therapist. Overall, we found a positive effect of music therapy on outcomes regarding psychological well-being, quality of life and physical symptom distress. With regard to different oncological treatment phases, the outcomes and techniques used in music therapy studies were heterogeneous. During curative cancer treatment, music therapy had a positive impact on well-being, anxiety, depression, mood, and pain, although findings were mixed. Interestingly, several studies showed a reduction of analgesic intake in response to music therapy in the course of surgery or transplantation. The interventions used in these treatment stages were mainly relaxation and imagery, singing, and improvisation with instruments that were feasible to play. Evidence on the effects of music therapy in aftercare is very scarce. Only one pilot study examined group music therapy after breast cancer treatment and reported positive effects on body image and feeling of identity. The validity of this finding, however, is very limited due to the extremely low sample size (Allen, 2010). In palliative populations, music therapy was successfully used to reduce anxiety and stress, and to improve spiritual and psychophysiological well-being. Findings with regard to pain reduction were mixed although the largest RCT provides promising evidence for a positive effect (Gutgsell et al., 2013). Studied techniques in these settings were either music-based relaxation or individually tailored interventions.

Considering the meta-analytical results, the effects of music therapy were small but significant for all three outcome categories. In comparison with previously reported findings (Archie et al., 2013; Bradt et al., 2016; Bro et al., 2018), the effect sizes in our meta-analysis were smaller. One reason might be the inclusion of similar outcomes in one category rather than one meta-analysis for each outcome which would have increased statistical bias due to multiple testing. Additionally, our effect size calculation method took pretest-discrepancies into account and is a more conservative method than often used posttest-comparisons (Morris and DeShon, 2002).

Heterogeneity between effect sizes was small to medium. With regard to quality of life and physical symptom distress, no moderators were found. Differences with regard to patients (inpatient /outpatient, curative/palliative treatment phase), treatment (active/receptive music therapy, duration, and frequency of sessions) or methodology (standard care vs. active control group) were not able to explain any variation in the calculated outcomes. This finding, however, does not necessarily lead to the conclusion that these and other moderating factors have no influence at all. The small number of effect sizes per outcome and the deliberate categorization of moderators may also contribute to the lack of significant findings. Moreover, other factors might be relevant which could not be monitored and tested in this analysis, such as pain medication or musical background. The moderators that explained variance between effect sizes of psychological well-being were session frequency and type of music therapy. Contrasting our expectations, studies that were limited to single sessions of music therapy produced more positive results than music therapy programs with higher session frequencies. It is possible that the first music therapy session in the work with chronically-ill cancer patients may induce strong reactions as music can instantaneously address feelings which the patient might not have been aware of. Moreover, studies with single-session music therapy often may have a less emotionally-challenging therapeutic focus, e.g., on relaxation or acute pain reduction (Gutgsell et al., 2013; Warth et al., 2015a). In an ongoing study on a biographic music therapy technique in palliative care (Warth et al., 2019b), we observe that the process of emotional and spiritual integration of past live events in some cases only starts to work after three or more sessions. Hence, the risk of wrong timing of the post-intervention assessment may be lower in single-session studies. The observed effect, however, might also be due to a methodological artifact, as the time span between pre and post assessment is very low in single-session studies and patients may remember their previous response to questionnaires. Moreover, receptive methods led to stronger improvements in psychological outcomes, which is consistent with previous meta-analytic findings on music therapy in dementia (Tsoi et al., 2018). One reason might be that active methods can be more challenging for patients due to insecurities or inhibition to express their feelings with music or simply because of physical weakness. Therefore, the immediate reaction of a patient to active music therapy might be less positive than the immediate reaction to receptive techniques. This is in line with Yates and Silverman (2015) describing that patients in their study tended to prefer receptive methods, especially in the first session.

In all three categories, exclusion of studies after sensitivity analysis led to smaller but still significant effect sizes. With regard to psychological well-being and physical symptom distress, the excluded studies were highly influential but also relevant because they had large sample sizes in comparison to the other studies (Gutgsell et al., 2013; Dóro et al., 2017; Rossetti et al., 2017). Regarding quality of life, the identified highly influential study was the only non-randomized controlled trial (Domingo et al., 2015) and therefore likely to be biased.

### Limitations

One of the key strengths of this review is the inclusion of only music therapy studies distinguishing the effects from other music interventions without a therapeutic relationship. Therefore, our conclusions can be applied for the evaluation and practical improvement of music therapy. Additionally, the categorization of effects in different oncological treatment phases constitutes a precise overview of the patients' needs in each phase and helps to design specific music therapeutic interventions for different cancer stages.

However, there are some limitations that should be considered for interpretation of results. First of all, seven studies could not be included in our meta-analyses due to insufficient data limiting the generalizability of our results. Second, risk of bias was high in all studies which may be due to the Cochrane tool for assessment originally developed for general randomized controlled trials. Even though it was adjusted for psychotherapy research (Munder and Barth, 2017), many studies performed poorly in this assessment. Another critical issue refers to the small number of studies included, especially in the meta-analysis on quality of life, as well as the small sample sizes of some studies. These factors might contribute to an underpowered analysis. Furthermore, due to missing evidence in the studies, we cannot draw conclusions about long-term effects of music therapy in psycho-oncology. As the number of sessions partially moderated the effects in our analysis, future studies are encouraged to employ longitudinal study designs to assess the long-term impact. With regard to moderators, interesting factors like experiences with and preferences of music (Bro et al., 2018) could not be taken into account because they are rarely assessed in studies. Still, as the individual relationship with music plays a key role in music therapy, further research should consider examining aspects of the patients' musical background, such as subjective value of music in one's life, years of musical and instrumental training and years of active musical participation, preference of musical style or prior amount of experience with music therapy, as potential modulating variables. Another possible influence which studies often do not report is pain medication. However, it could be an interesting control variable as cancer patients especially in palliative care settings often receive high pain medication and its effect might be confounded with the effect of music therapy on pain.

## Conclusion

Considering the results of the present systematic review and meta-analysis, we found mixed, but overall positive and significant effects of music therapy on psychological well-being, quality of life and physical symptom distress in different phases of oncological treatment. As the diagnosis and treatment of cancer is often accompanied by challenging physical symptoms and psychological distress, even small improvements through music therapy may be relevant for patients with oncological diseases. With regard to research, studies on music therapy are encouraged to reduce risk of bias, e.g., through publishing primary and secondary outcomes in a study protocol, and to assess long-term effects of music therapy. In addition, potential moderators should be included in the measurements, such as individual information about patients, to find out who benefits most from different kinds of music therapy and to provide techniques that fit the individual needs of a patient.

## Author Contributions

FK, Z-SM, and MW contributed to conception of the review, organized database, performed statistical analysis, and wrote the first draft of the manuscript. FK, Z-SM, R-SH, CG, BD, JK, and MW revised the work critically. All authors approved the submitted version.

### Conflict of Interest

The authors declare that the research was conducted in the absence of any commercial or financial relationships that could be construed as a potential conflict of interest.
