# Corrigendum: Music Therapy in the Psychosocial Treatment of Adult Cancer Patients: A Systematic Review and Meta-Analysis

**DOI:** 10.3389/fpsyg.2020.02095

**Published:** 2020-09-18

**Authors:** Friederike Köhler, Zoe-Sofia Martin, Ruth-Susanne Hertrampf, Christine Gäbel, Jens Kessler, Beate Ditzen, Marco Warth

**Affiliations:** ^1^Institute of Medical Psychology, Center for Psychosocial Medicine, University Hospital Heidelberg, Heidelberg, Germany; ^2^Ruprecht-Karls University Heidelberg, Heidelberg, Germany; ^3^Department of Communication and Psychology, Aalborg University, Aalborg, Denmark; ^4^Center of Pain Therapy and Palliative Care Medicine, Department of Anesthesiology, University Hospital Heidelberg, Heidelberg, Germany

**Keywords:** music therapy, oncology, cancer, effectiveness, randomized controlled trials, quality of life, supportive care, complementary therapies

In the original article, there was a mistake in Table 2 as published. The “Setting and patients” description of the study by Rossetti et al. (2017) should read “Outpatient medical center, patients with breast or head and neck cancer, *N* = 78” instead of “Inpatient medical center, patients with breast, head or neck cancer, *N* = 78”. The “Intervention” description of the study by Rossetti et al. (2017) should read “Music therapy (multiple techniques); 1 session, 60 min” instead of “Music listening; 1 session, 60 min.” The corrected Table 2 appears below.

**Table 2:** Study characteristics.

**Study**	**Setting and patients**	**Intervention**	**Control group**	**Study design**	**Main findings**	**Outcomes included in meta-analysis**
Alcântara-Silva et al. (2018)	Outpatient cancer treatment hospital, patients with gynecological cancer, *N* = 164	Music listening; 10 sessions, 35 min	TAU	RCT	Stronger increase in quality of life, stronger decrease in fatigue and depression in EG	
Allen (2010)	After care, patients with breast cancer who were in remission, *N* = 11	Music imaging (group therapy); 10 sessions, 60 min	ACG: cognitive behavioral therapy	RCT	Stronger increase in feelings of identity, family role relationship, self-esteem and body image in EG	
Bates et al. (2017)*	Inpatient blood and marrow transplantation unit, patients with lymphoma or myeloma, *N* = 82	Music listening and creating; 2 sessions, 30 min.	TAU	RCT	Less pain medication in EG, no significant difference in subjective pain perception	PSYCH: POMS
Bieligmeyer et al. (2018)*	Inpatient oncology department, patients with breast or colorectal cancer, *N* = 44	Sound bed; 1 session, 15 min	TAU	RCT	Stronger increase in well-being, quality of life and physiological symptoms in EG, no significant differences in pain and social extraversion	PSYCH: BMQ, PHYSIC: VAS
Bradt et al. (2015)*	In and outpatients from a hospital, patients with cancer (various types), *N* = 31	Music therapy (multiple techniques); 2 sessions, 20–45 min	ACG: listening to music	RCT	No significant group differences in mood, anxiety, relaxation and pain	PSYCH: VAS, PHYSIC: 11-point numeric rating scale
Burns (2001)*	Outpatient oncology offices, patients with breast or ovarian cancer, *N* = 8	Bonny method of guided imagery and music; 10 sessions, 60–90 min	Waitlist	RCT	Stronger increase in mood and quality of life in EG	PSYCH: POMS, QOL: QOL-CA
Burns et al. (2007)	Inpatient hematology oncology unit, patients with leukemia	Music imagery; 8 sessions, 45 min	TAU	RCT	No significant group difference in positive and negative effect, anxiety and quality of life	
Burns et al. (2018)	Outpatients from cancer centers, patients with various types of cancer, *N* = 86	Guided visualization with music; 1 session, 45–50 min	ACG: listening to music	RCT	Stronger increase in responsiveness and benefit finding in EG, stronger decrease in distress in CG	
Cassileth et al. (2003)*	Inpatient cancer centers, patients with lymphoma or myeloma, *N* = 69	Music listening and creating; 3–7 sessions, 20–30 min.	TAU	RCT	Stronger decrease in depression, anxiety and mood in EG	PSYCH: POMS
Chen et al. (2018)	Outpatient medical center, patients with breast cancer, *N* = 52	Music imagery; 8 sessions, 60 min	TAU	RCT	Stronger decrease of depression, helplessness, hopelessness and cognitive avoidance in EG	
Cook and Silverman (2013)	Inpatient oncology-hematology unit, patients with leukemia and other cancers, *N* = 34	Music listening and conversations; 3 sessions, 15–30 min	Waitlist	RCT	Stronger increase in spiritual well-being in EG	
Domingo et al. (2015)*	Inpatient palliative care unit, patients with advanced cancer, *N* = 68	Music therapy (multiple techniques); 4 sessions, 30*40 min	TAU	CCT	Stronger increase in well-being in EG	PSYCH: HADS, QOL: well-being single item
Dóro et al. (2017)*	Inpatient allogeneic hematopoietic stem cell transplantation unit, patients with neoplastic hematologic disorders, *N* = 100	Music singing and improvisation; 8 session, 30 min	TAU	RCT	Stronger increase in mood in EG, no significant difference in anxiety and pain	PSYCH: VAS; PHYSIC: VAS
Fredenburg and Silverman (2014)*	Inpatient adult blood and marrow transplantation unit, patients with leukemia and lymphoma, *N* = 11	Music therapy (multiple techniques); 30–45 min	Waitlist	RCT	No significant group difference	PHYSIC: MFI
Fredenburg and Silverman (2014)	Inpatient adult blood and marrow transplantation unit, patients with leukemia, lymphoma and myeloma, *N* = 32	Music therapy (multiple techniques); 1 sessions, 30 min	Waitlist	RCT	Stronger decrease of pain in EG, stronger increase in negative and positive effect in EG	
Gutgsell et al. (2013)*	Inpatient medical center, patients with advanced cancer (26 non-cancer patients), *N* = 200	Music relaxation; 1 session, 20 min	Waitlist	RCT	Stronger decrease in pain in EG	PHYSIC: 11-point numeric rating scale
Hanser et al. (2006)*	In and outpatients from breast oncology clinic, patients with breast cancer, *N* = 42	Music therapy (multiple techniques); 3 session, 45 min	TAU	RCT	Increase in relaxation, comfort and happiness in EG	QOL: FACT-G, PHYSIC: FACT-G (subscale), PSYCH: HADS
Hilliard (2003)*	Outpatient hospice, patients with advanced cancer, *N* = 80	Music therapy (multiple techniques); 2–13 sessions	TAU	RCT	Stronger increase in quality of life in EG, no significant difference in length of life or physical functioning	PSYCH: HQLI-R, QOL: HQLI-R
Horne-Thompson and Grocke (2008)*	Inpatient palliative care unit, patients with advanced cancer (1 non-cancer patient), *N* = 25	Music therapy (multiple techniques); 1 session, 20–40 min	ACG: volunteer visit	RCT	Stronger decrease in anxiety	PSYCH: ESAS, PHYSIC: ESAS
Letwin and Silverman (2017)*	Inpatient medical oncology/hematology unit, patients with various types of cancer, *N* = 15	Music listening; 2 sessions, 30–45 min	Waitlist	RCT	No significant group difference in resilience and pain	PSYCH: RSES, PHYSIC: 11-point numeric rating scale
Lin et al. (2011)*	Inpatient medical center/hospital, patients with various types of cancer, *N* = 98	Music imagery; 1 session, 60 min	TAU	RCT	Stronger decrease in anxiety in EG, stronger increase in skin temperature in EG	PSYCH: STAI
Palmer et al. (2015)*	Inpatient university hospital, patients with (potential) breast cancer, *N* = 201	Music listening before and during surgery; 1 session	TAU	RCT	Stronger decrease in anxiety and faster recovery after surgery in EG	PSYCH: VAS
Porter et al. (2018)*	Inpatient palliative care unit, patients with advanced cancer (4 non-cancer patients), *N* = 42	Music therapy (multiple sessions); 2–6 sessions, 45 min	TAU	RCT	Stronger increase in well-being	QOL: MQoL, PSYCH: MQoL, PHYSIC: MQoL
Ramirez et al. (2018)	Inpatient palliative care, patients with advanced cancer, *N* = 40	Music relaxation, active and receptive songs; 1 session, 30 min	ACG: Conversation about music	RCT	Stronger increase in valence and arousal and well-being in EG	
Rossetti et al. (2017)*	Outpatient medical center, patients with breast or head and neck cancer, *N* = 78	Music therapy (multiple techniques); 1 session, 60 min	TAU	RCT	Stronger decrease in anxiety and distress in EG	PSYCH: STAI
Tuinmann et al. (2017)*	Inpatient medical center, patients with lymphoma, *N* = 66	Music playing, singing and listening; 8 session, 20 min.	TAU	RCT	Stronger decrease in need of analgesics and subjective pain perception in EG	QOL: EORTC QLQ-C30, PAIN: EORTC QLQ-C30
Verstegen (2016)*	Inpatient blood and marrow transplantation unit, patients with cancer, *N* = 10	Music listening and therapeutic dialogue; 2 session, 30–60 min	TAU	RCT	Stronger increase in hope in EG, no significant difference in pain	PSYCH: HHI, PHYSIC: 11-point numeric rating scale
Wang et al. (2015)	Inpatient cancer hospital, patients with lung cancer, *N* = 60	Music relaxation during surgery, music listening afterwards; 5 sessions, 60 min	TAU	RCT	Stronger decrease in anxiety, lower blood pressure and heart rate, less need for analgesics in EG	
Warth et al. (2015b)*	Inpatient palliative care, patients with cancer (2 non-cancer patients), *N* = 84	Music relaxation; 2 sessions, 30 min	ACG: pre-recorded mindfulness exercise	RCT	Stronger increase in relaxation and well-being and in high-frequency oscillation of the heart rate and stronger decrease in fatigue in EG, no significant difference in pain	QOL: VAS, PSYCH: EORTC QLQ C15-PAL, PHYSIC: VAS
Yates and Silverman (2015)*	Inpatient surgical oncology unit, patients with colon/rectal or uterine cancer, *N* = 26	Music listening; 1 session, 20–30 min	Waitlist	RCT	Stronger decrease in anxiety and increase in relaxation in EG	PSYCH: QMS

Studies marked with * were included in meta-analyses; TAU, treatment as usual; ACG, active control group; RCT, randomized controlled trial; CCT, controlled clinical trial; EG, experimental group; CG, Control group; PSYCH, psychological well-being; QOL, quality of life; PHYSIC, physical symptom distress; Poms, Profile of mood states; BMQ, Basler Mood Questionnaire; VAS, Visual Analogue Scale; QOL-CA, Quality of life – Cancer scale; HADS, Hospital Anxiety and Depression Scale; MFI, Multidimensional Fatigue Inventory; FACT-G, Functional Assessment of Cancer Therapy—General; HQLI-R, Hospice Quality of Life Index—Revised; ESAS, Edmonton Symptom Assessment Scale; RSES, Response to Stressful Events Scale; STAI, Stait Trait Anxiety Inventory; MQoL, McGill Quality of Life Questionnaire; EORTC QLQ-C30, Quality of Life Questionnaire; HHI, Herth Hope Index; EORTC QLQ-C15-Pal, Quality of Life Questionnaire for Palliative Care; QMS, 12-item quick mood scale.

In the original article, there was a subsequent error in the description of the intervention in the study by Rossetti et al. (2017).

A correction has been made to “Results,” “Study Description and Narrative Synthesis,” “Music Therapy During Chemotherapy and Radiation”, Paragraph 2:

“Two studies assessed the effects of music therapy in the course of radiation therapy and found reductions in anxiety and distress (Rossetti et al., 2017) as well as improvements regarding quality of life, fatigue, and depression (Alcântara-Silva et al., 2018). Techniques used in these studies encompassed listening to prerecorded music, live music therapy, and conversations with a therapist.”

The authors apologize for these errors and state that they do not change the scientific conclusions of the article in any way. The original article has been updated.
